# Cubic-meter scale laboratory fault re-activation experiments to improve the understanding of induced seismicity risks

**DOI:** 10.1038/s41598-022-11715-6

**Published:** 2022-05-15

**Authors:** Volker Oye, Sergey Stanchits, Oladipupo Babarinde, Robert Bauer, Anna Maria Dichiarante, Nadège Langet, Bettina Goertz-Allmann, Scott Frailey

**Affiliations:** 1 NORSAR, Gunnar Randers vei 15, 2007 Kjeller, Norway; 2 Skolkovo Institute of Science and Technology, Bolshoy Boulevard 30, bld. 1, Moscow, 121205 Russia; 3 Illinois State Geological Survey, Prairie Research Institute, University of Illinois at Urbana-Champaign, 615 E. Peabody Drive, Champaign, IL 61820 USA

**Keywords:** Seismology, Geophysics, Energy storage, Characterization and analytical techniques

## Abstract

To understand fluid induced seismicity, we have designed a large-scale laboratory experiment consisting of a one-cubic-meter sandstone with an artificial fault cut and fluid-injection boreholes. The sandstone block is assembled in a true triaxial loading frame and equipped with 38 piezoelectric sensors to locate and characterise acoustic emission events. The differential stress on the artificial fault is increased in stages to bring it towards a critically stressed state. After each stage of differential stress increase, fluids are injected at low pressures through boreholes to test the potential of fault re-activation. In addition, a high-pressure injection was conducted that created a hydraulic fracture from the injection borehole towards the artificial fault. The newly generated fluid pathway resulted in an activation of the complete block through a stick–slip movement. We compare acoustic emission measurements from the laboratory experiment with seismicity observations from the field-scale CO_2_ injection at Decatur, Illinois, U.S., and conclude that the existence of fluid pathways plays a decisive role for the potential of induced seismicity.

## Introduction

Carbon Capture and Storage (CCS) is one of the necessary mitigation technologies to combat and limit global climate change. Verification of the containment and seal integrity of large-scale geological CO_2_ reservoirs requires a suite of geophysical monitoring technologies. Among these, microseismic monitoring, i.e. listening to tiny crackles arising from minuscule rock movements, can identify potential activation of unwanted fluid pathways early enough to ensure storage site integrity and initiate mitigation measures in a timely manner. High-pressure fluid injections that exceed the fracture pressure of the rock can create fractures and open fluid pathways, which is often desired to enhance fluid flow in geothermal developments^1^ or in shale gas fracking^2^. In contrast, CO_2_ injections are carried out at much lower pressures, as fracture opening is to be avoided: the CO_2_ is supposed to stay in place for hundreds to thousands of years. However, brittle deformation also occurs during CO_2_ injection operations, e.g. along pre-existing faults and fractures. These can be reactivated already by very small pore pressure or stress changes, if they are critically stressed and favourably oriented with respect to the stress field.

Microseismic activity by fault reactivation has been observed in a number of CCS pilot projects over the last decade. At the In Salah, industrial-scale CO_2_ storage project in Algeria, low-level seismicity occurred at distances of up to 3–4 km from the CO_2_ injection well, in proximity to a pre-existing fault. This seismicity appeared unrelated to injection pressures and injection rates, but was nevertheless interpreted as a sign of reactivation of the mapped fault, either due to large-scale reservoir deformation or through injection-related stress field changes^3,4^. The triggered seismicity could be further constrained to the seal, i.e. about 150 m above the reservoir^3^. It is exactly these types of events that real-time monitoring needs to reliably identify such that mitigating actions can be taken.

Deformation along pre-existing faults and fractures—though not in the seal—was observed at the Illinois Basin—Decatur Project (IBDP), where over one million tonnes of CO_2_ were injected over a period of three years, starting in 2011^5,6^. Over these three years of injection, about 5000 microseismic events were located within roughly 2.5 km distance from the injection well (Fig. ES1). Interestingly, seismicity occurred both within the reservoir formation and in the basement directly beneath. Again, the seismicity does not directly correlate to injection parameters, measured and modelled pressure evolution, or estimated CO_2_ saturation front movement, but a general causality with the CO_2_ injection is evident^6^. In both Decatur and In Salah, microseismicity is strongly clustererd spatially. A high-resolution monitoring network at Decatur allowed to further reveal clear linear structures within the microseismic event clusters^7^ and focal mechanisms^8^, which are correlating with the preferred fracture orientation arising from the known regional stress regime in the basement^9,10^. A potential explanation for the reactivation of critically stressed fault patches in the basement is pressure communication through basement faults^11,12^. This explanation is consistent with a reduction in seismic activity at Decatur, when injection was shifted towards a shallower injection well, positioned above some shale baffles which are acting as barriers to fluid flow towards the basement faults^10^.

The phenomenon of fault reactivation was also studied with observations and experiments in underground research labs where dedicated drill-holes within mining sections and tunnels were made available for controlled fluid-injection experiments at the decameter-scale. Monitoring of deformation, microseismicity and acoustic emissions with various instruments were e.g. conducted in shales at Mt Terri, Switzerland^13^, carbonates at Tournemine, France^14^ and within granites in a Swedish mine by Zang et al.^15^. These in-situ experiments have the advantage that the reactivated structure can be clearly identified, and its parameters, including the pore pressure, can be determined with more certainty compared to field-scale experiments^16^. In the underground research labs, most commonly the fault reactivation by fluid injection above fracture pressure was studied, with injection directly into damage zones and also with differently phased injection durations and pressures to investigate relaxation damage^15^. Similar fault reactivation as a result of injection above the fracture pressure have also been observed in geothermal developments^17^, as well as during shale gas fracking^18^.

In order to reconcile observed fault reactivation during high-pressure injections with seismicity occurring along pre-existing fractures at much lower injection pressures around CCS sites, we conducted a laboratory experiment^19^. The experiment is designed to study how low-pressure fluid injection can cause seismicity along pre-existing faults at a given distance to the injection. The exposure of a rock sample to true triaxial loading is here combined with controlled fluid injection rates and pressures, as well as measurements of deformation and acoustic emissions. Acoustic emission (AE) experiments are primarily conducted to test rock strength and failure mechanisms at various scales and under different boundary conditions^20–24^. For example, in a pioneering study of stick–slip sliding of artificial interfaces with 2 m length induced by water injection, Lockner et al.^25^ measured the values of dynamic stress drop, interface slip and slip velocities, allowing to better understand the mechanics that ultimately lead to unstable failure. Advances over the last decade allowed testing of larger samples under true triaxial stress conditions^26^, and incorporating fluid pressure control on the sample^27^. High-pressure fluid injections into samples led for example to an improved understanding of hydraulic fracturing in shale gas environments^28,29^. Improvements towards smaller sensor sizes allowed for an increasing number of piezoelectric sensors to be mounted around the sample, which significantly improved the AE location accurracy, and allowed for characterisation of source types and moment tensor inversions^30,31^. Measurements with calibrated acoustic emission sensors on samples with artificial faults^32^ have observed seismic events with absolute magnitudes as low as − 6. These appear to be a scaled version of large earthquakes, hence supporting the self-similarity hypothesis of brittle deformation^33,34^.

We first present the setup of the laboratory experiment specifically designed to observe the response of a critically stressed fault to fluid-injection. We then present the results of the various phases of the experiment followed by a discusion of the implications towards CCS injection operations, where we link the characteristically different types of AE-energy releases observed in the laboratory experiment with field scale observations such as the ones from In Salah and Decatur.

## Experimental setup for the acoustic emission monitoring and fluid injection

**Figure 1:**
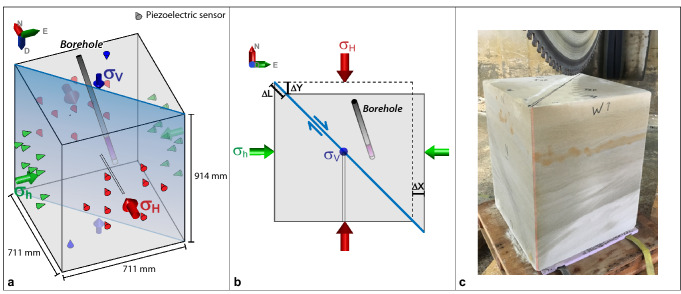
Picture and schematics of the experimental setup. (**a**) Schematic 3D-view of the assembled block showing the positions of the 38 piezoelectric sensors (red, green and blue cones), the main injection borehole, a secondary injection borehole reaching into the interface and the saw cut interface (blue plane). Green, red, and blue arrows show applied stresses. (**b**) Plan-view of the block indicating measured displacements ∆x and ∆y and calculated interface displacement ∆L. The open section of the main injection borehole is indicated with magenta shading. (**c**) Picture of the Castlegate Sandstone block just before cutting the interface.

To design an experiment on potential fault reactivation caused by fluid injection through a borehole at some distance, we opted for the currently largest available loading frame with true triaxial loading capabilities, while fully capturing 3D effects and instrumenting all 6 loading plates with AE sensors. As the experimental design is motivated by the observed seismicity at Decatur, we selected a sandstone sample that is a representative analogue to the Lower Mt. Simon reservoir Sandstone, with similar permeability, porosity and originating from fluvial deposition. Also, practical aspects such as availability at the required size after cutting (711 × 711 × 914 mm), safe transport from the quarry to the laboratory and associated costs all led towards selecting the well-known Castlegate Sandstone as analogue rock. In preparation of the large-block experiment, we simulated a pre-existing fracture in a much smaller, cylindrical loading frame of 38.1 mm diameter and 76.2 mm height, also using Castlegate Sandstone from the same quarry. Here, the shear strength of the rock was first exceeded until brittle failure started, and then the differential stress was lowered, and pore pressure in the whole sample increased until the freshly created fracture was reactivated^35–37^. Because of the technical limitations of the large loading frame, it was not possible to fracture the intact sandstone block. Therefore, we created a saw cut to split the block diagonally in two equal halves (see Fig. 1c and photographs in ES2), then laser-scanned both surfaces and reassembled the block halves again, now consisting of an interface created by the saw cut of about 1 m length and 914 mm height^38^. To control the fluid injection pressure, a borehole was drilled with an open hole section of 152 mm length and at distance of 108 mm from the block interface (Fig. 1a,b). Pore pressures were continuously monitored at the injection borehole and through a second injection borehole reaching directly into the interface (Fig. 1a,b). The loading frame directly measured the block deformation along each direction, and 38 single-component piezoelectric sensors were embedded in the loading plates of all six faces of the block to record acoustic emissions at 2.5 MHz sampling rate^39^ (Fig. 1a). This procedure is analogous to the recording and analysis of microseismicity at field scale.

**Figure 2:**
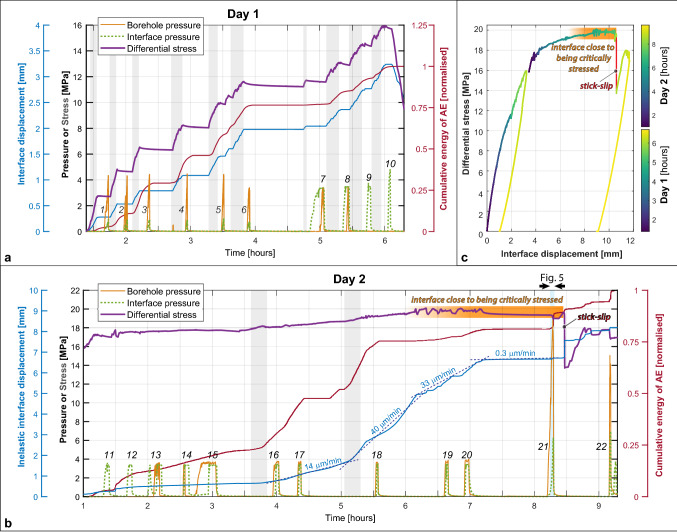
Overview of the 2-day testing of the Castlegate Sandstone block. (**a**) Day-1 of the AE experiment with incremental increases of the differential stress (gray shading), related release of AE energy, interface displacement/creep and borehole/interface fluid injection pressures. Injection-stages 1–10 are indicated by numbers. (**b**) Day-2 of the experiment. Gray shading shows two time intervals where differential stresses were increased by about 0.4 and 0.5 MPa, respectively, both times accompagnied and directly followed by an increase in AE activity. The interface creep rate increased to about 30–40 μm/min, until the maximum differential stress was reached and the interface continued to creep with only 0.3 μm/min. The blue shading shows the time interval of the high-pressure fluid injection, followed by related fracture creation and stick–slip movement of the whole block. (**c**) Interface displacement vs differential stress for entire experiment, day 1 stops after 6 h. Note that (**a**) and (**c**) show interface displacement, whereas (**b**) shows inelastic interface displacement in order to visualize the related displacement at the time of the stick–slip.

The 2-day test procedure contained these main steps (Fig. 2a,b): (1) hydrostatic loading up to 15 MPa (σ_V_ = σ_h_ = σ_H_); (2) injection into the interface of 26 l of chemically inert silicone oil of 0.04 Pa·s viscosity to provide saturation of most parts of the block, including the interface section; (3) during day 1 of the test, we kept the maximum horizontal stress constant at σ_H_ = 15 MPa, while decreasing σ_h_ and σ_V_ stepwise down to 3.4 MPa; (4) on day 2, we then kept σ_h_ and σ_V_ at 3.4 MPa and further increased the differential stress ∆σ by increasing σ_H_ stepwise up to 23.4 MPa (Fig. 2, Fig. ES3); (5) throughout the 2 days, fluid injection tests were conducted at both boreholes to investigate AE-trigger potential.

In total, 22 steps were performed to adjust the differential stress, and following each of the steps, fluid injection tests were performed with silicone oil of 0.04 Pa·s and 0.2 Pa·s viscosity with up to 4.5 MPa injection pressure in the offset injection borehole. Our hypothesis was that fluid injection far below the fracture pressure of the Castlegate Sandstone (about 18 MPa) would be able to induce AE activity at the offset interface and reactivate the whole interface to slide, i.e. trigger a laboratory equivalent to an earthquake. At the end of the experiment (step 21), the borehole injection pressure was increased up to 18.5 MPa in order to hydraulically fracture the region around the injection borehole.

## Results

Throughout the 2-day laboratory experiment, we observed three distinctively different phases of block sliding behaviour and we will describe them in the following sections (1) *from a locked interface to aseismic creep*, (2) *fracture creation and fracture opening and, (3) stick–slip interface movement*.

### From a locked interface to aseismic creep

After the block was hydrostatically loaded and partially saturated, the stepwise increases in the differential stress directly resulted in minor interface sliding of the two sides of the block relative to each other, accompanied by an increase of acoustic emission energy attributable to grain crushing and breaking of minor asperities or contacts within the interface, supported by AE event locations, analysis of laserscans before and after the experiment, and clay mineral compositional analysis of the created gouge material^38^. The AE activity related to each of the stages decreased as soon as the new level of differential stress was reached (Fig. 2a,b). During the first six stages, fluids were injected through the offset borehole for about one to three minutes durations increasing up to about 4.5 MPa, which caused a slight increase in pore pressure at the interface of about 1 MPa. However, the rate of AE activity continued to be decreasing after each incremental increase in the differential stress and no increase in the occurrence rate of AEs was observed during the injection phases, not at the interface and neither around the injection borehole. For the stages 7–8, the injection pressure was in addition also raised in the injection borehole reaching directly into the interface, and during stages 9 and 10 the injection was only conducted through the interface borehole. None of these injections did provoke any increase in the occurrence rate of AE energy, and together with the quasi-elastic relation between interface displacement vs differential stress as depicted in Fig. 2, indicates that the interface was not critically stressed yet. This concluded the first day of the experiment without fluid injection-related AEs, even though the differential stress was increased up to 15.9 MPa.

During day 2, the differential stress was then further raised by increasing the largest horizontal stress up to 23.4 MPa, reaching a maximum differential stress of 20 MPa at about hour 6 of day 2, from where on the interface is close to being critically stressed (Fig. 2b,c). None of the short periods of fluid injections of day 2 at about 4.5 MPa injection pressure, either directly towards the interface or through the offset borehole did cause any change on the interface displacement or on the rate of released AE energy. At differential stresses of about 18 MPa (or about 4 h 30 min in day 2), we start to observe an onset of interface displacement that we characterize as stable fault creep (Fig. 2b,c). At first with a rate of ~ 14 μm/min, then at ~ 40 μm/min, and slowing down to about 33 μm/min. Shortly after 7 h, the differential stress was slightly lowered from the maximum of 20 MPa to about 19.4 MPa, resulting in a stable, though much reduced fault creep of only 0.3 μm/min.

**Figure 3:**
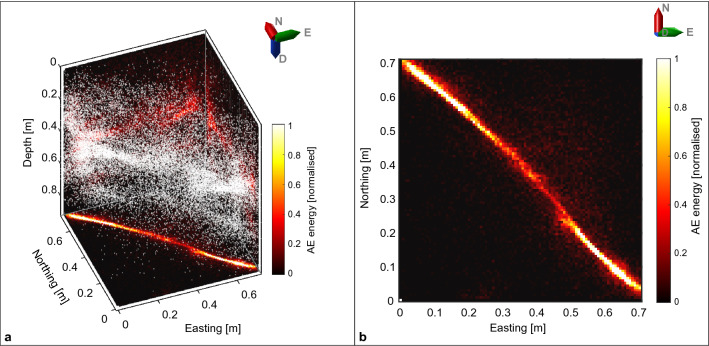
AE events of the first 20 stages of the experiment preceding the high-pressure injection. (**a**) 3D-view of the block with individual event locations (white dots) and color-coded cumulative AE energy of all located events. (**b**) Plan-view of the cumulative AE energy. Most of the events are located right at the interface, with slightly less energy originating from the central part of the interface.

**Figure 4:**
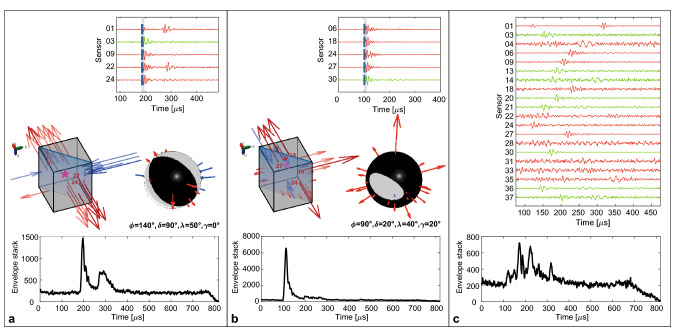
Characteristics of AE-events. (**a**) Shear-type event. Commonly observed during first 20 stages of the experiment, located along interface. Selected waveforms (green and red traces) correspond to AE-sensor-locations on σh and σH faces of the block. Block diagram shows event location (star) and arrows with first-motion amplitudes at the sensor locations and corresponding full moment tensor solution. The bottom plots show envelope stacks aligned along P-wave onsets. The shear type event shows clear energy for P and S waves. (**b**) Tensile-opening event related to the fracture creation under high-pressure injection. The envelope stack shows only one energy accumulation. (**c**) Events during stick slip movement show continuous increase in amplitudes on all traces, with significant increase in the background noise level. Individual phases are difficult to identify, likely multiple events occur simultaneously and only the largest event is located.

Seismicity during the first 20 low-pressure injection stages is dominated by repeated stress redistributions along the interface and occurrence of AEs as a response to increased differential stresses and periods of creep. The located AE events originate primarily right at the interface, without any specific temporal evolution, though spread over the whole interface (Fig. 3). Most events show both P- and S-wave energy, often containing clear phase onsets (Fig. 4) that have been identified through automatic methods, visually confirmed and if required manually adjusted, e.g.^40^*.* Due to the large number of sensors (38) distributed around all faces of the block, location uncertainties of individual AE events are generally only in the order of 2–4 cm, small velocity model variations from start to end of experiment also influence the location precision slightly, but do not impact the general observations. Moreover, focal mechanism analysis of selected events results primarily in strike-slip solutions (Fig. 4), in accordance with the observed interface creep, whereas some of the smaller events away from the interface are likely due to small pore collapse as a response to stress increase^24^. The event locations in Fig. 3 deviate slightly from the interface, which is likely an artifact due to the homogeneous velocity model used in the event location, even though the fluid injection introduced an area of faster velocities around the injection area.

### Fracture creation and fracture opening

**Figure 5:**
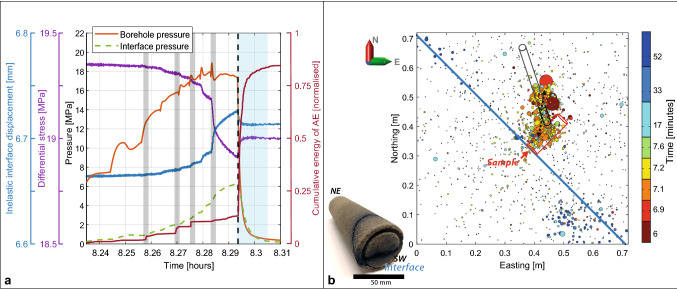
High-pressure fluid injection and fracture creation. (**a**) Increasing borehole pressure results in several high-energy AE-events (steps in cumulative AE energy, grey shaded areas depict the largest individual AE events) of primarily tensile-opening character (Fig. 4b). Shortly after reduction of the injection pressure (blue shaded area), a cascade of AE events occurred, with primarily mixed-mode and closing mechanisms. (**b**) AE event locations showing temporal evolution of the cloud from the injection point developing a bi-wing fracture both towards and away from the interface (see black dashed arrows). Size of events is related to AE-energy. Bottom left shows the rock sample drilled from the interface into the newly created fracture, showing blue ink where the fracture was identified.

None of the low-pressure injection stages did influence the character of the interface displacement or increase the AE-activity level. Therefore, we decided to apply injection pressures that hydraulically fractured the stressed Castlegate Sandstone by injecting fluid through the main injection point at a pressure of about 18 MPa (Fig. 5a), five times above the applied minimum horizontal stress *σh*, ~ 3.4 MPa. Already during the upramping of the injection pressure from about 14 to 16 MPa, first high-energy AE events with clear fracture opening source mechanism were identified (Fig. 4b). The location of these first events concentrate around the open section of the injection borehole, and the moment tensor analysis is based on first motion polarities, amplitudes and amplitude ratios^8^. The generation of a bi-wing fracture increased the permeability around the injection borehole and allowed more fluid to the fault interface. Following the pressure release at the injection borehole, a large sequence of AE events were recorded focusing around the area of the newly created fracture of about 20 cm height, connecting the borehole with the interface (Fig. 5b). After the experiment, samples were drilled out of the sandstone block and the newly created fracture was identified and coincided with the locations of the AE event cloud of primarily tensile source mechanism (Figs. 4, 5b). Throughout the high-pressure fluid injection in the borehole creating the hydraulic-fracture and the following sequence of AE events between the borehole and the interface, no significant AE events occurred on the interface, and only about 0.04 mm displacement occurred on the interface during the high-pressure injection phase, where the measured pressure at the interface was temporarily reaching 6.2 MPa, even though no injection was conducted through the interface.

### Stick–slip interface movement

After the high-pressure injection that created the bi-wing fracture, an enhanced permeability path was created linking the borehole injection to the interface, allowing to transport the injected fluids more effectively towards the interface. About 10 min after the pressure in the injection borehole was lowered, the differential stress was again slightly increased by about 0.5–19.6 MPa, which was below the previously applied maximum differential stress of 20 MPa and at that time did result in a steady creep of about 30–40 μm/min. This time though, the two sides of the block were suddenly and unstably sliding relative to each other with an offset of about 0.8 mm interface displacement, and accompanied by a loud and clearly recognized “thwomp” to the engineers present in the laboratory, a stick–slip event, or an earthquake at laboratory scale.

**Figure 6:**
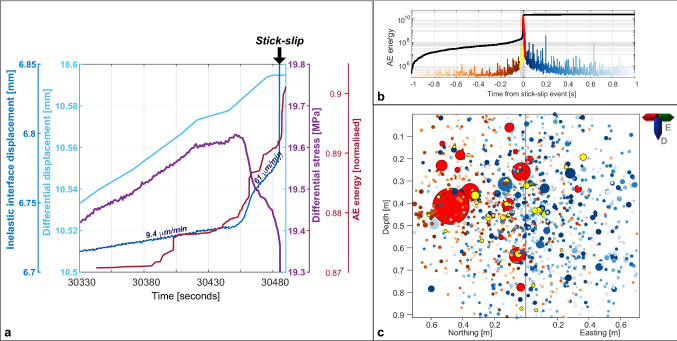
Evolution of stick–slip event. (**a**) Inelastic and differential interface displacements show an acceleration phase of the inelastic displacement only 10 s before the initiation of the stick–slip event. Differential stresses start to decrease about 30 s before the stick–slip, and continue to decrease also during the last 10 s before the stick–slip, even at constant differential displacement, expressing slip-weakening behavior of the interface. (**b**) Stacked AE-energy plotted along 2 s centered around the main-shock of the stick–slip event. Y-axis is logarithmic in scale and shows increase of about 5 orders of magnitude in AE-energy from noise level 1 s before the stick–slip and the main shock. Note that the energy level after the stick–slip is generally higher than before. Applied colorcode is also valid to individual events located along the interface in (**c**), showing temporal evolution of events along the interface, indicating movement from one to the other side. The size of the circles relates to the energy of the events in logarithmic scale.

Investigating the AE waveform data before, during and after the stick–slip movement, we did not observe any significant number of AE events based on conventional detection methods for transient signals. However, applying a migration-based waveform stacking method based on^41^ on two seconds of data around the actual stick–slip event, we did observe a clear and gradual increase in the AE-energy or tremor-like noise starting from about 20 to 40 ms before the stick–slip event (Figs. 4, 6). Even before these pre-cursor noise tremors, we did observe an accelerated interface movement of about 60 μm/min, starting already about 35 s before the stick–slip occurred (Fig. 6a). This accelerated interface slip rate is an average estimate as we can see some slip rate variability. As such it is noteworthy that the differential stress has reached the maximal value and started decreasing about 30 s before the stick–slip event, and continues to decrease even though the externally applied displacement of the block boundaries remain constant within the last 5–10 s before the stick–slip event occurs. This behaviour is often described as slip-weakening^42^, when the friction coeffient is reduced due to slip or increased slip velocity at a fault. In this case, the newly created fracture at the injection borehole enabled additional access of fluids towards the interface, allowing higher fluid saturation and consequently reducing the normal stress across the interface, potentially explaining the slip-weakening behaviour that finally resulted in the stick–slip event. The AE energy emitted during this stick–slip main-shock is about 2 orders of magnitude larger than any of the aftershocks, and about 5 orders of magnitude larger than the noise level during the stable creeping period before the stick–slip (Fig. 6b).

Applying the migration-based approach on the two seconds of AE-data^41^, we were also able to show that the main tremor of AEs was initiated by foreshocks increasing in size and frequency of occurrence at one side of the block, preceeding the main stick–slip event with about 20 ms. This main event occurred close to the centre of gravity of the foreshocks, and immediate events after the main event migrate towards the other side of the block, where also most of the aftershock activity occurred. The distance between the main stick–slip event and the immediate first aftershock is about 40 cm, and they occurr about 1 ms after each other (Fig. 6). Assuming that these events connect with each other, a maximum rupture velocity of 400 m/s can be assumed, which is relatively low compared with natural earthquakes of about 1000–4000 m/s as compiled by e.g. Chounet et al.^43^. However, considering the almost 1 m^3^ size of the sandstone block, this rupture velocity likely represents the very first nucleation and acceleration phase of an earthquake-size rupture. We could also estimate the slip velocity of the sample from the absolute displacement during the stick–slip of 0.8 mm, and the estimated duration of the burst of AE events around the stick–slip main shock with a total duration of about 40 ms. This relates to an approximate slip velocity of about 0.02 m/s, which is within the range of estimates for earthquakes (0.001–10 m/s) according to Rowe and Griffith^44^. We also provide a rough magnitude estimate of the stick–slip movement, simply by computing the seismic moment *M*_*0*_ based on the displacement *D*, the estimated slip area *A* and the rigidity μ of the Castlegate Sandstone: *M*_*0*_ = *D·A·*μ, assuming a maximum of 0.8 mm for D, 0.25 m as source radius for A = πr^2^ (estimated from Fig. 6) and about 6 GPa for μ. This results in a maximum estimate for the moment magnitude *M*_*w*_ of − 2.1 and a lower estimate of *M*_*w*_ − 2.7 assuming only 0.4 mm for the displacement D and 0.125 m for the source radius.

## Discussion

**Figure 7:**
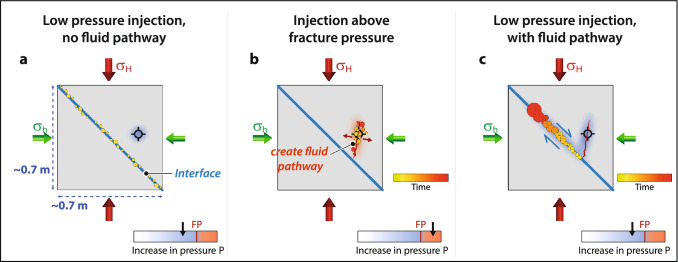
Schematics of three characteristic types of seismicity as observed in the laboratory experiment and analogue field cases. (**a**) Increasing differential stress on interface results in loading-related seismicity on interface. Low pressure fluid injection at an offset injection borehole does not influence seismicity nor creep at the interface as fluid pathways are not connected. (**b**) High-pressure injection creates a hydraulic fracture opening and generates a new pathway for fluids to enter the interface. (**c**) Saturation at interface is increased, and stick–slip movement initiates cascade of AE events moving the whole block or induce a large earthquake.

The laboratory experiment was originally designed based on the hypothesis that stress-field changes related to low-pressure fluid injection may be sufficient to trigger seismic activity on critically stressed faults. The hypothesis was motivated by observations at several CCS injection sites where low magnitude microseismic events were observed in relation to CO_2_ injection. The laboratory results show that stepwise loading of the differential stress resulted in prompt AE event activity along various patches of the interface, and this activity slowly decreased again once the differential stress remained constant. In contrast to our hypothesis, none of the low-pressure fluid injections at a distance to, or right into the interface were able to initiate increased seismic activity or significant interface displacement, even when the interface was critically stressed (Fig. 7a). This leads us to suggest that CO_2_ storage sites that do not have any fluid pathways towards critically stressed faults, and where the injection pressure is controlled and stays below the formation fracture pressure, are not expected to experience induced seismicity as a consequence of low-pressure fluid injection.

In cases where the injection pressure is raised above the formation fracture pressure, microseismic events are expected to occur, creating and extending hydraulic fractures and fluid pathways. The first events are often associated with fracture opening, whereas later microseismic events often show mixed focal mechanisms, including fracture closing type events (Fig. 7b). This kind of microseismicity generally eases over time after the injection pressure is reduced. This was for example observed at the Krechba, In Salah CO_2_ injection project, where the microseismic activity related to high-pressure injection decreased significantly within hours to days^3^, and this is also commonly observed in geothermal and shale-gas operations^18,45^.

One challenge in hydraulic fracture operations is that high-pressure injections may also generate new fluid pathways that lead to or connect with pre-existing fractures or faults. We observed this behaviour in the laboratory experiment, where the newly created fracture opened a fluid pathway to the interface and a laboratory-scale earthquake was induced (Fig. 7c). At field-scale, similar cases were observed e.g. at the Pohang geothermal stimulation site in South Korea, where a magnitude M_w_ 5.5 earthquake was triggered about two months after the injection was stopped^46^. Similarly, at the Krechba, In Salah CCS site, low-magnitude seismicity continued to occur at a nearby fault without correlation to CO_2_ injection pressures or rates^3^. Once fluid conduits are existing, the injection pressure may not be a relevant parameter to predict induced seismicity. In these cases, even low-pressure injections can result in clusters and bursts of seismicity. At Decatur, a network of pre-existing open basement faults is suspected to provide a means for triggering seismicity in clusters at a distance to the injection well^9^.

Early identification of potential precurser events or onset of aseismic creep before any large earthquake might nucleate has long been searched for. In underground research labs some evidence for aseismic creep preceding seismic events has been discussed by e.g. Guglielmi et al.^47^ at the deca-meter scale. Our laboratory experiment at the meter-scale was able to identify an onset of accelerated creep about 30 s before the stick–slip event occurred. Furthermore, we were able to locate an increase of tremor-like AE events about one second before and at the same location as the main stick–slip event. This kind of observation is likely impossible to use for any practical earthquake warning approach, however, new detection methods and instrumentation can be tested at laboratory scales to advance the science in this field.

In conclusion, we suggest from our results and comparison between laboratory experiments and field-scale observations, that upfront fracture mapping and dedicated continuous monitoring of microseismicity will allow to assess and potentially mitigate risks for induced seismicity at any large-scale fluid injection operation, be it waste-water, CO_2_, hydrogen, or natural gas storage. Sudden changes in fluid pathways are often preceeded by seismicity, and if these are identified in due time, the positions of potentital new clusters of seismicity may be discovered early enough to conduct risk evaluations for induced seismicity or even leakage potential.

## Methods

### Experimental setup and computation of block deformation

In the experiment, the two halves of the block were placed in a polyaxial loading frame made by TerraTek, a Schlumberger Company. The stress frame can apply a maximum vertical stress of 55 MPa, and horizontal stresses of up to 41 MPa with a maximum differential stress of 27 MPa can be applied independently. After drilling of the boreholes, they were cased for stability and to controle the injection. For the main injection borehole, we used steel tubing with a 25.4 mm outer diameter. The tubing was glued into the borehole using a flexible, two-component epoxy adhesive. To reduce stress concentrations along the bottom-hole edge, a 25.4 mm epoxy plug was added to the bottom of the borehole. In completion, the open-hole section between the glued tubing and the plug was ~ 152.4 mm long. The center of the open-hole section was located approximately 108 mm away from the sliding interface. The second wellbore of 12.7 mm diameter was drilled in the opposing block half from the center-point of the face directly to the center-point of the sliding interface. Steel tubing was glued in this second wellbore using the same two-component epoxy through its entire length.

To reduce the shear stresses on the block surfaces we placed Teflon sheets between the loading plates and the faces of the rock. In order to assure good mechanical coupling, the AE sensors were embedded in the loading plates and were in direct contact with the rock surface through holes in the Teflon sheets. We did not conduct a full calibration of the individual AE sensors with respect to their absolute amplitudes and directional sensitivity, including potential frequency dependencies. However, some tests using individual sensors as piezoelectric sources showed generally balanced coupling of the sensors, and polarities of the sensors were consistent and quality controlled. However, we normalized all computations of AE energies in this manuscript.

In this experiment we wanted to avoid any potential influence due to chemical processes like stress corrosion on the interface sliding and we therefore selected silicone oil, a chemically inert fluid. Due to the above-mentioned Teflon sheets, the boundaries of the block were not fully sealed, and parts of the injected fluids might flow out of the block through the outer faces of the block. This was another reason why we chose the silicone oil as injection agent, as the higher viscosity helped avoid leakage of the fluid through the faces of the block and also to achieve significant pore pressure increase within the block and the interface.

A true triaxial loading frame allows three principal stresses to be applied to the block by using flat jacks with an accurate measurement of the volume of fluid injected to pressurize each flat jack. The results of these volumetric measurements in each direction were normalized to the area of the corresponding faces of the block to calculate the block displacements: *Δx*—along *σ*_*h*_ direction and *Δy*—along *σ*_H_ direction (Fig. 1b). Taking into account a diagonal cut of the block at an angle of 45°, the displacement *ΔL* along the interface was calculated as follows: $\Delta L=\sqrt{2}(\Delta y-\Delta x)/2$ and differential stress as: σ_d_ = σ_H_ − σ_h_. We separate the total interface displacement *ΔL* into an elastic component $\Delta {L}_{Elastic}$ and an inelastic component $\Delta {L}_{Inel}$, assuming that the inelastic interface displacement $\Delta {L}_{Inel}= \Delta L- \Delta {L}_{Elastic}$ (based on the general approach of Scholz^48^). Based on the analysis of the block deformation data, we found that $\Delta {L}_{Inel} = \Delta L-\left(1.0796+0.00096885\cdot \sigma d\right)$. The relationship between the elastic interface displacement $\Delta {L}_{Elastic}$ and the differential stress *σ*_*d*_ was determined based on the analysis of the initial part of the loading curve presented in Fig. 2C, assuming that at the beginning of the loading, in the range covering differential stresses from 3.3 until 9.8 MPa, the fault was completely locked, and the total deformation of the block was elastic. The coefficient of proportionality between the elastic interface displacement $\Delta {L}_{Elastic}$ and the differential stress *σ*_*d*_ is related to the compressibility of the fluid inside the flat jacks and to the stiffness of the sandstone.

## Supplementary Information

Supplementary Information 1.

Supplementary Information 2.

Supplementary Information 3.

Supplementary Information 4.

## Data Availability

The AE-waveform data as well as location data will be provided through NORSAR’s data repository at https://doi.org/10.21348/d.no.0002.

## References

[1] Zang A (2014). Analysis of induced seismicity in geothermal reservoirs—an overview. Geothermics.

[2] Maxwell SC, Rutledge J, Jones R, Fehler M (2010). Petroleum reservoir characterization using downhole microseismic monitoring. Geophysics.

[3] Goertz-Allmann BP, Kühn D, Oye V, Bohloli B, Aker E (2014). Combining microseismic and geomechanical observations to interpret storage integrity at the In Salah CCS site. Geophys. J. Int..

[4] Vasco DW (2018). Monitoring and modeling caprock integrity at the In Salah carbon dioxide storage site, Algeria. Geol. Carbon Stor. Subsurf. Seals Caprock Integrity.

[5] Bauer RA, Carney M, Finley RJ (2016). Overview of microseismic response to CO2 injection into the Mt Simon saline reservoir at the Illinois Basin-Decatur Project USA. J. Greenh. Gas Sci. Technol..

[6] Bauer RA, Will R, Greenberg SE, Whittaker SG, Davis T, Landrø M, Wilson M (2019). Illinois Basin-Decatur Project, Chapter 19. Geophysics and Geosequestration.

[7] Dando BDE (2021). Relocating microseismicity from downhole monitoring of the Decatur CCS site using a modified double-difference algorithm. Geophys. J. Int..

[8] Langet N (2020). Joint focal mechanism inversion using downhole and surface monitoring at the Decatur, Illinois, CO2 injection site. Bull. Seismol. Soc. Am..

[9] Dichiarante AM (2021). Identifying geological structures through microseismic cluster and burst analyses complementing active seismic interpretation. Tectonophysics.

[10] Williams-Stroud S (2020). Analysis of microseismicity and reactivated fault size to assess the potential for felt events by CO2 injection in the Illinois Basin. Bull. Seismol. Soc. Am..

[11] Goertz-Allmann BP, Gibbons SJ, Oye V, Bauer R, Will R (2017). Characterization of induced seismicity patterns derived from internal structure in event clusters. J. Geophys. Res. Solid Earth.

[12] Goertz-Allmann, B. P. *et al*. Long-term seismic monitoring of reservoir dynamics at decatur. In *Proceedings of the 15th Greenhouse Gas Control Technologies Conference. *10.2139/ssrn.3820454 (2021).

[13] Guglielmi Y, Birkholzer J, Rutqvist J, Jeanne P, Nussbaum C (2017). Can fault leakage occur before or without reactivation? Results from an in situ fault reactivation experiment at Mont Terri. Energy Proced..

[14] Guglielmi Y (2021). Field-scale fault reactivation experiments by fluid injection highlight aseismic leakage in caprock analogs: Implications for CO2 sequestration. Int. J. Greenh. Gas Control.

[15] Zang A (2021). Relaxation damage control via fatigue-hydraulic fracturing in granitic rock as inferred from laboratory-, mine-, and field-scale experiments. Sci. Rep..

[16] Zang A, Stephansson O (2010). Stress Field of the Earth’s Crust.

[17] Kim KH (2018). Assessing whether the 2017 Mw 5.4 Pohang earthquake in South Korea was an induced event. Science.

[18] Maxwell, S. C*., et a*l. Fault activation during hydraulic fracturing. In *SEG Technical Program Expanded Abstracts 2009*, 1552–1556. Society of Exploration Geophysicists. 10.1190/1.3255145 (2009).

[19] Frailey, S. M. *et al*. Center for the Geologic Storage of CO_2_ (GSCO2) (Final Report). United States: N. p. 10.2172/1691497 (2020).

[20] Lockner D, Byerlee JD, Kuksenko V, Ponomarev A, Sidorin A (1991). Quasi-static fault growth and shear fracture energy in granite. Nature.

[21] Lockner D (1993). The role of acoustic emission in the study of rock fracture. Int. J. Rock Mech. Min. Sci. Geomech. Abstr..

[22] Ishida T (2017). ISRM suggested method for laboratory acoustic emission monitoring. Rock Mech. Rock Eng..

[23] Gori M, Rubino V, Rosakis AJ, Lapusta N (2021). Dynamic rupture initiation and propagation in a fluid-injection laboratory setup with diagnostics across multiple temporal scales. Proc. Natl. Acad. Sci..

[24] Manthei G, Zang A, Grosse CU, Grosse CU, Ohtsu M, Aggelis DG, Shiotani T (2022). Laboratory acoustic emission in study of rock mechanics. Acoustic Emission Testing. Springer Tracts in Civil Engineering.

[25] Lockner DA, Okubo PG, Dietrich JH (1982). Containment of stick slip failures on a simulated fault by pore fluid injection. Geophys. Res. Lett..

[26] Nasseri MHB, Goodfellow SD, Lombos L, Young RP (2014). 3-D transport and acoustic properties of Fontainebleau sandstone during true-triaxial deformation experiments. Int. J. Rock Mech. Min. Sci..

[27] Stanchits S, Burghardt J, Surdi A (2015). Hydraulic fracturing of heterogeneous rock monitored by acoustic emission. Rock Mech. Rock Eng..

[28] Stanchits, S., Desroches, J., Burghardt, J., Surdi, A. & Whitney N. Rock fabric influence on hydraulic fracture propagation. In *77th EAGE Conference and Exhibition 2015*, Madrid, Spain, 1–4 June 2015. Conference Paper Tu-N107-01. 10.3997/2214-4609.201412632 (2015b).

[29] Suarez-Rivera, R. *et al*. Defining three regions of hydraulic fracture connectivity, in unconventional reservoirs, help designing completions with improved long-term productivity. In *Proceedings-SPE Annual Technical Conference and Exhibition*, 7, Conference Paper, 5049–5062. 166505-MS SPE. 10.2118/166505-MS (2013).

[30] Vera Rodriguez I, Stanchits S (2017). Spatial and temporal variation of seismic attenuation during hydraulic fracturing of a sandstone block subjected to triaxial stress. J. Geophys. Res. Solid Earth.

[31] Vera Rodriguez I, Stanchits S, Burghardt J (2017). Data-driven, in-situ, relative sensor calibration based on waveform fitting moment tensor inversion. Rock Mech. Rock Eng..

[32] McLaskey GC, Kilgore BD, Lockner DA, Beeler NM (2014). Laboratory generated M-6 earthquakes. Pure Appl. Geophys..

[33] Abercrombie RE (1995). Earthquake source scaling relationships from− 1 to 5 ML using seismograms recorded at 2.5-km depth. J. Geophys. Res. Solid Earth.

[34] Allmann BP, Shearer PM (2009). Global variations of stress drop for moderate to large earthquakes. J. Geophys. Res. Solid Earth.

[35] Stroisz, A. M. *et al*. Monitoring of fracture reopening in sandstones. In *50th US Rock Mechanics/Geomechanics Symposium* (2016)

[36] Cerasi P, Holt RM, Lavrov A, Stenebråten JF (2016). Investigation of geomechanical and rock physics aspects related to underground storage and monitoring of CO_2_. J. Ind. Geophys. Union.

[37] Cerasi P (2018). Experimental investigation of injection pressure effects on fault reactivation for CO_2_ storage. Int. J. Green. Gas Control.

[38] Babarinde O (2018). Analysis of injection-induced slippage in a large sandstone block via laser scanning, acoustic emissions, and pore pressure changes with stress. AGU Fall Meet. Abstr..

[39] Oye, V., *et al*. Dynamics of Stick-Slip Sliding Induced by Fluid Injection in Large Sandstone Block. In *80th EAGE Conference and Exhibition 2018* (Vol. 2018, No. 1, pp. 1–5). European Association of Geoscientists & Engineers. 10.3997/2214-4609.201800718 (2018).

[40] Oye V, Roth M (2003). Automated seismic event location for hydrocarbon reservoirs. Comput. Geosci..

[41] Gharti HN, Oye V, Roth M, Kühn D (2010). Automated microearthquake location using envelope stacking and robust global optimization. Geophysics.

[42] Olsen-Kettle (2008). Analysis of slip-weakening frictional laws with static restrengthening and their implications on the scaling, asymmetry, and mode of dynamic rupture on homogeneous and bimaterial interfaces. J. Geophys. Res..

[43] Chounet A, Vallée M, Causse M, Courboulex F (2018). Global catalog of earthquake rupture velocities shows anticorrelation between stress drop and rupture velocity. Tectonophysics.

[44] Rowe CD, Griffith WA (2015). Do faults preserve a record of seismic slip: A second opinion. J. Struct. Geol..

[45] Deichmann N, Giardini D (2009). Earthquakes induced by the stimulation of an enhanced geothermal system below Basel (Switzerland). Seismol. Res. Lett..

[46] Shapiro SA, Kim KH, Ree JH (2021). Magnitude and nucleation time of the 2017 Pohang Earthquake point to its predictable artificial triggering. Nat. Commun..

[47] Guglielmi Y, Cappa F, Avouac JP, Henry P, Elsworth D (2015). Seismicity triggered by fluid injection–induced aseismic slip. Science.

[48] Scholz CH (1968). The frequency-magnitude relation of microfracturing in rock and its relation to earthquakes. Bull. Seismol. Soc. Ame..

