# Supplementary figures and images for: Cubic-meter scale laboratory fault re-activation experiments to improve the understanding of induced seismicity risks
Source: Sci Rep. 2022 May 15;12:8015. doi: 10.1038/s41598-022-11715-6 (PMC9108097; doi:10.1038/s41598-022-11715-6)

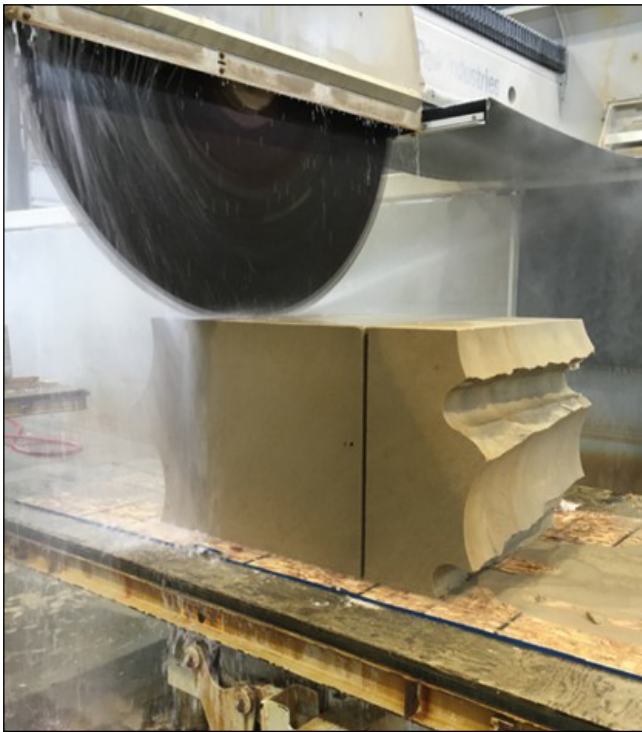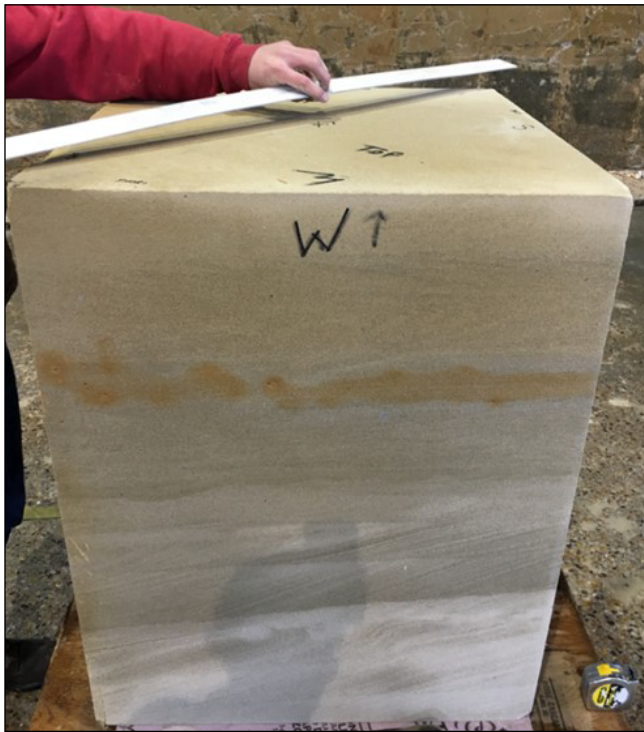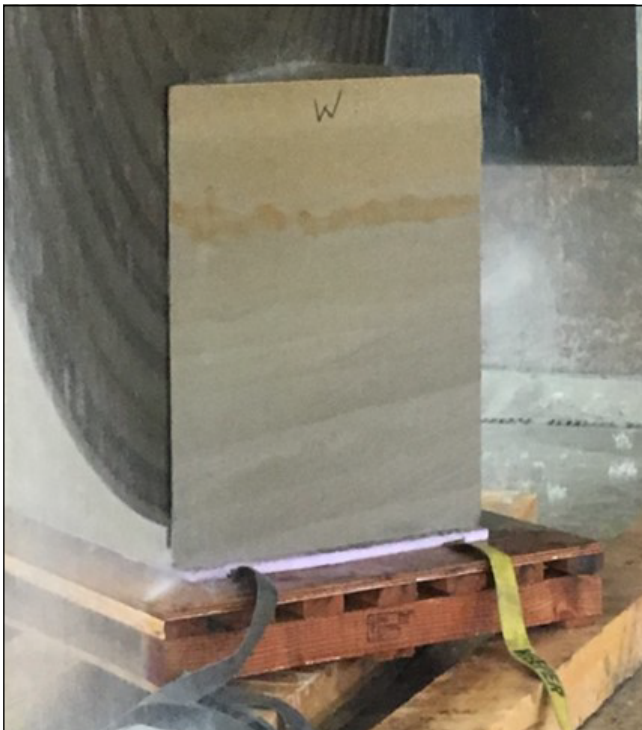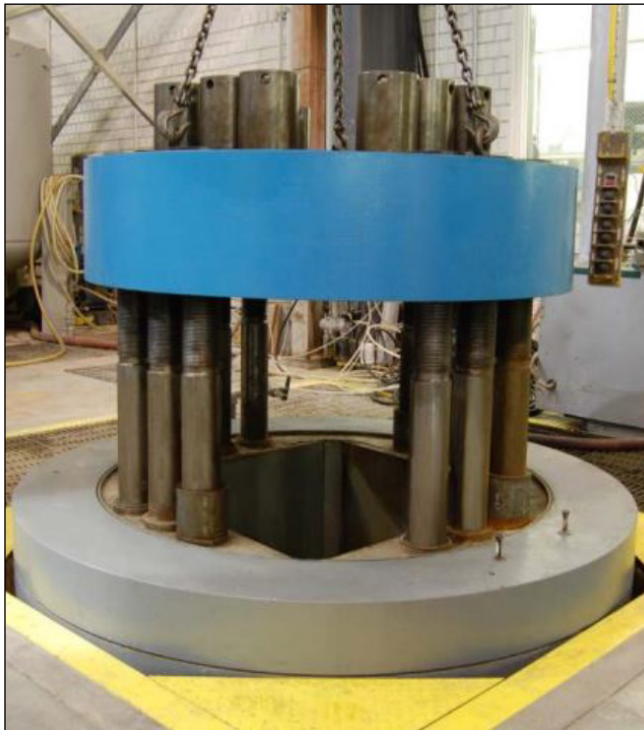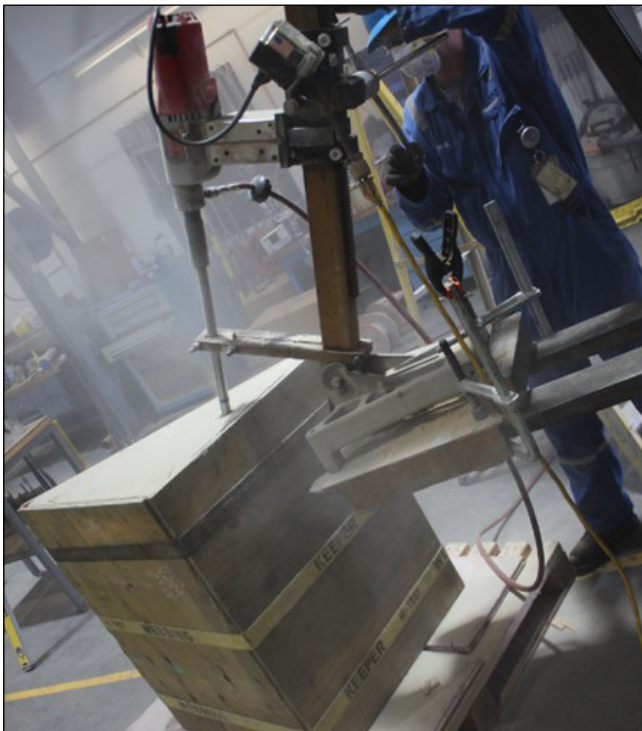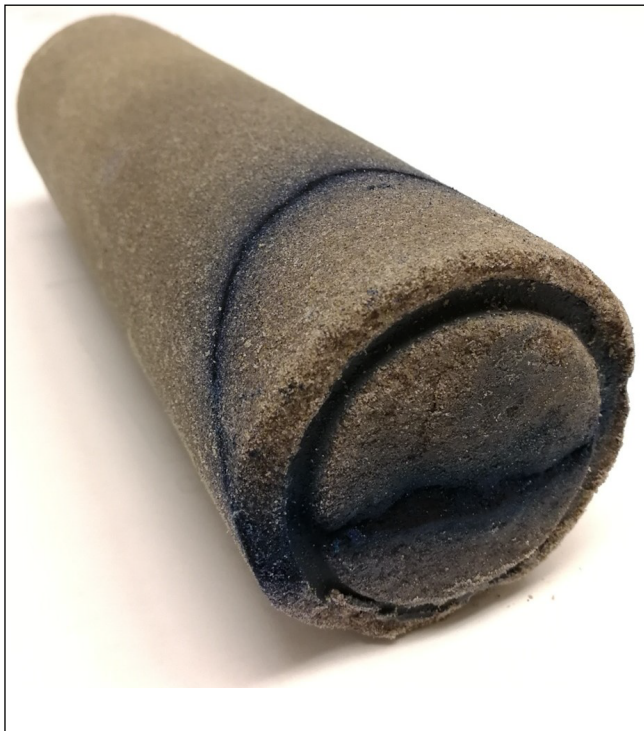

Supplement: Supplementary file 2 — Supplementary Information 2. [file 41598_2022_11715_MOESM2_ESM.pdf]

# Day 1

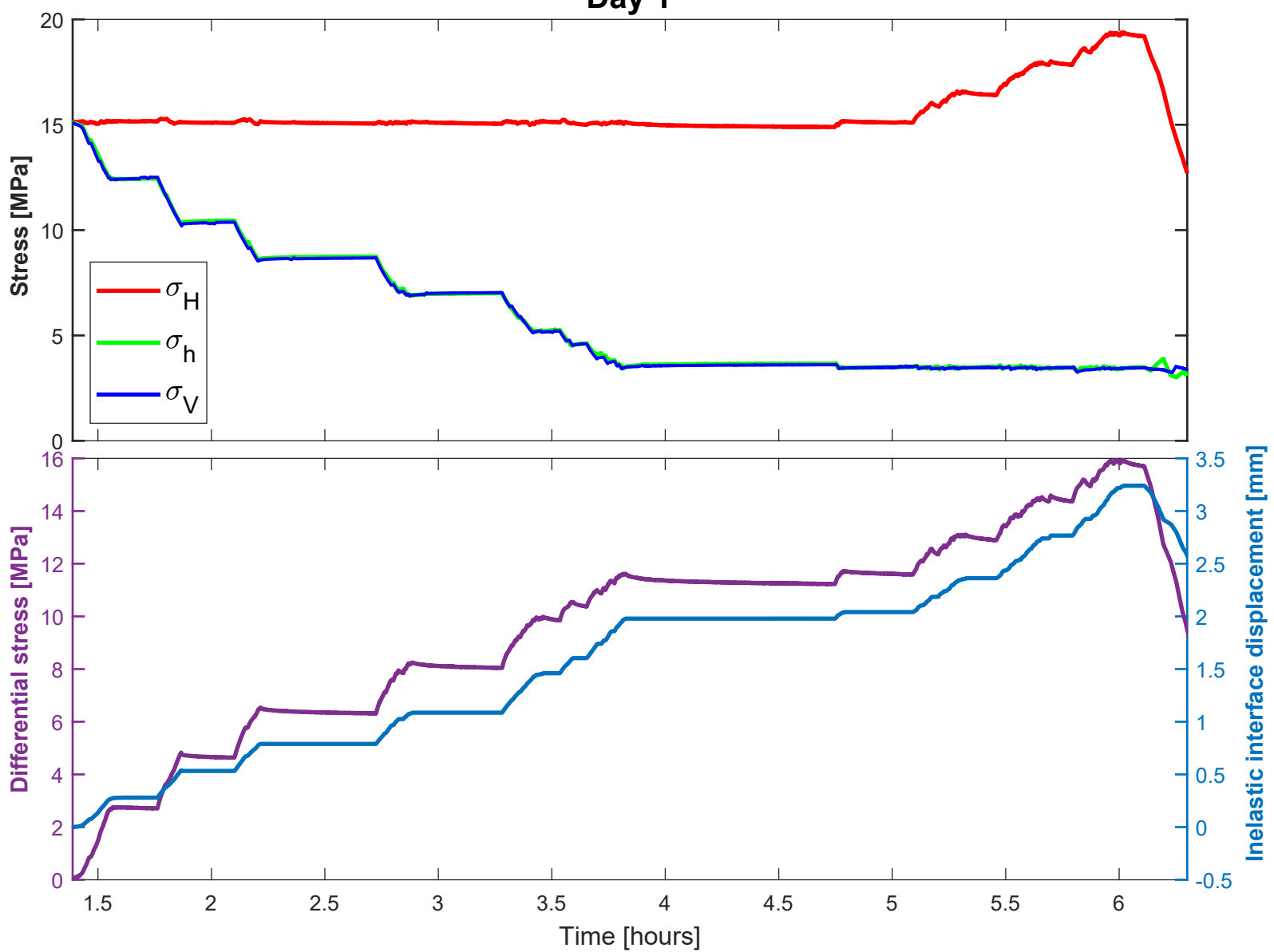

# Day 2

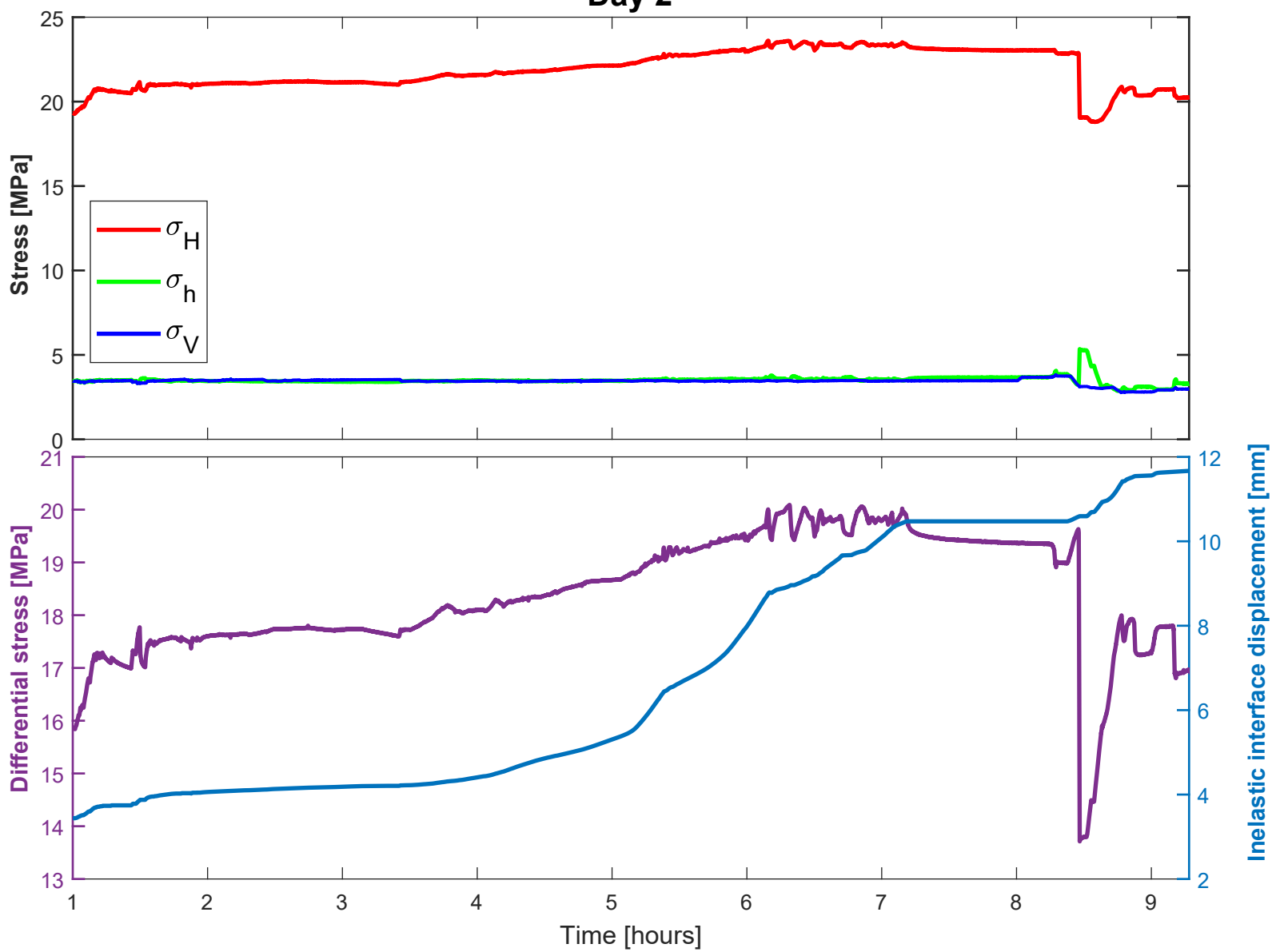

Supplement: Supplementary file 3 — Supplementary Information 3. [file 41598_2022_11715_MOESM3_ESM.pdf]
