# Supplementary material for: Cubic-meter scale laboratory fault re-activation experiments to improve the understanding of induced seismicity risks
Source: Sci Rep. 2022 May 15;12:8015. doi: 10.1038/s41598-022-11715-6 (PMC9108097; doi:10.1038/s41598-022-11715-6)
Supplement: Supplementary file 4 — Supplementary Information 4. [file 41598_2022_11715_MOESM4_ESM.pdf]

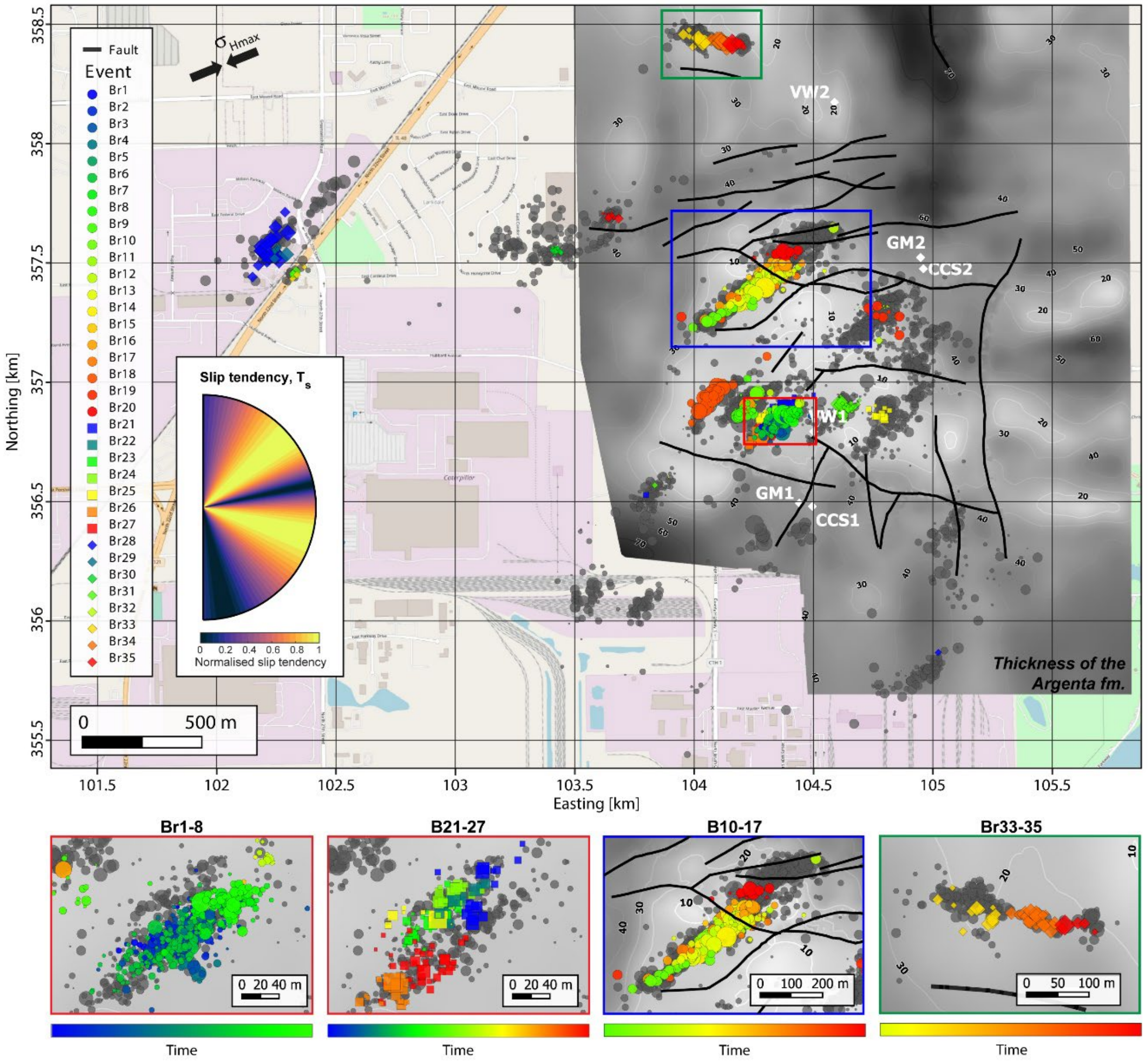

Figure ES1: *Map view of CO_2 injection related seismicity at Decatur. CO_2 was injected through CCS1 and CCS2 wells (white diamonds) during two injection phases, and microseismic events were located mostly into the Precambrian basement, with some events at reservoir depth and in the Argenta fm (formation between the reservoir and the underlying basement). Magnitude of seismicity is scaled by dot-size, largest event is M_W 1.6 [12]; Colored events represent 35 short temporal sequences (bursts, Br). (bottom) Close-up of three regions where consecutive bursts are observed. These events follow spatio-temporal patterns (see relative time scale) and interpreted to be activated by access to fluids [9]. Faults (black lines) are interpreted at the top of the basement on reprocessed 3D seismic data [10]. Greyscaled basemap modified from [10], showing thickness of Argenta fm, with isolines from almost 0 to about 70 m. Drilling-induced tensile fractures and breakouts in CCS1, CCS2, and two verification wells (VW1 and VW2, white diamond) indicate an average maximum horizontal stress (σ_{Hmax}) of N 68° E (arrows) without change in direction with depth (inset map). Normalized slip tendency (T_s) is shown for directions between 0°-180°. The overall geometry of some of the further distant clusters (e.g., far west cluster) shows higher slip tendency (closer to being a critically stressed fault segment) and have been activated with only minor stress-field changes.*

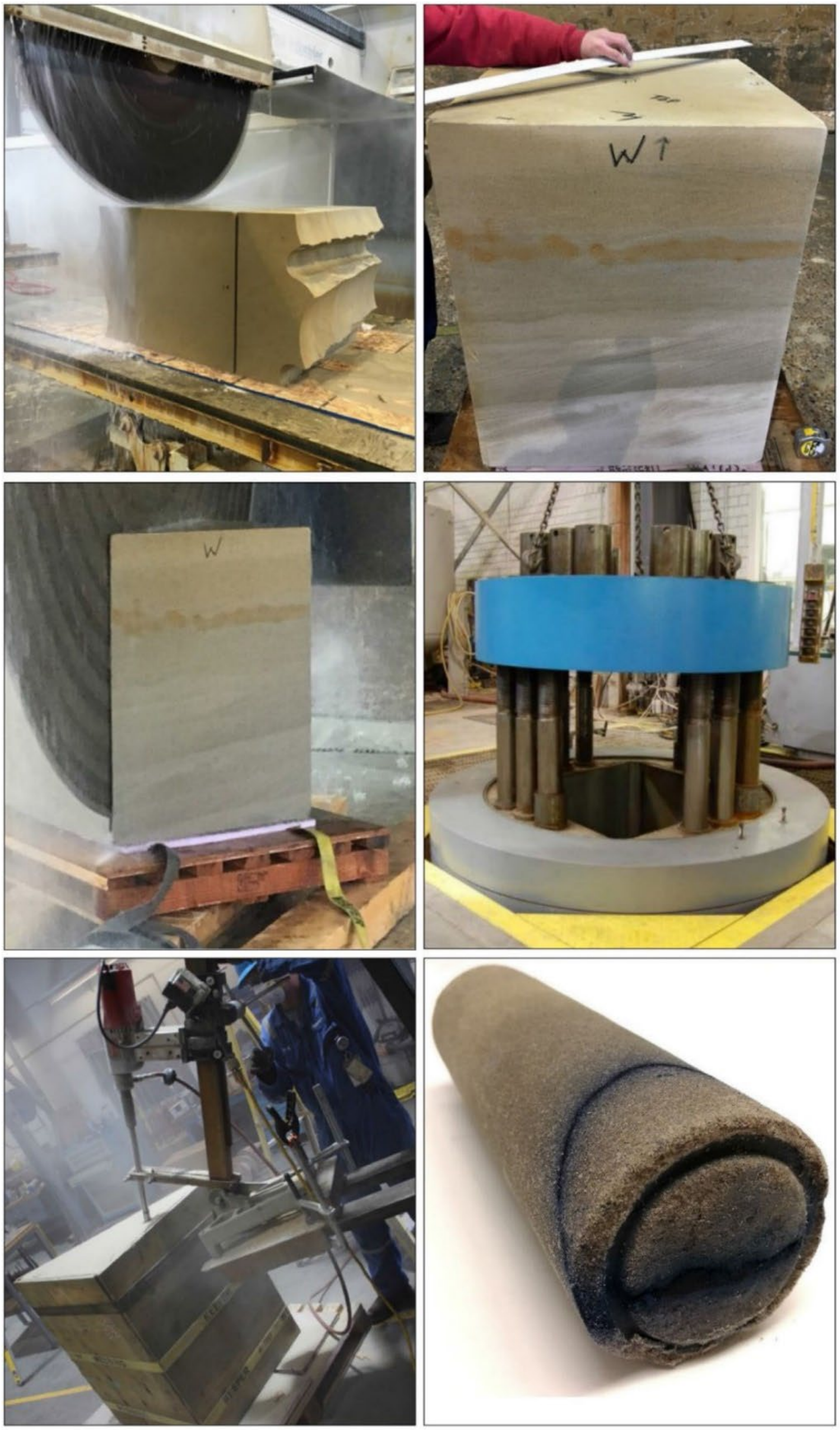

Fig. ES2: *Photographs from the loading frame, the sawing of the block and preparation of the boreholes and a high-resolution figure of the sample taken from the interface into the created hydrofracture.*

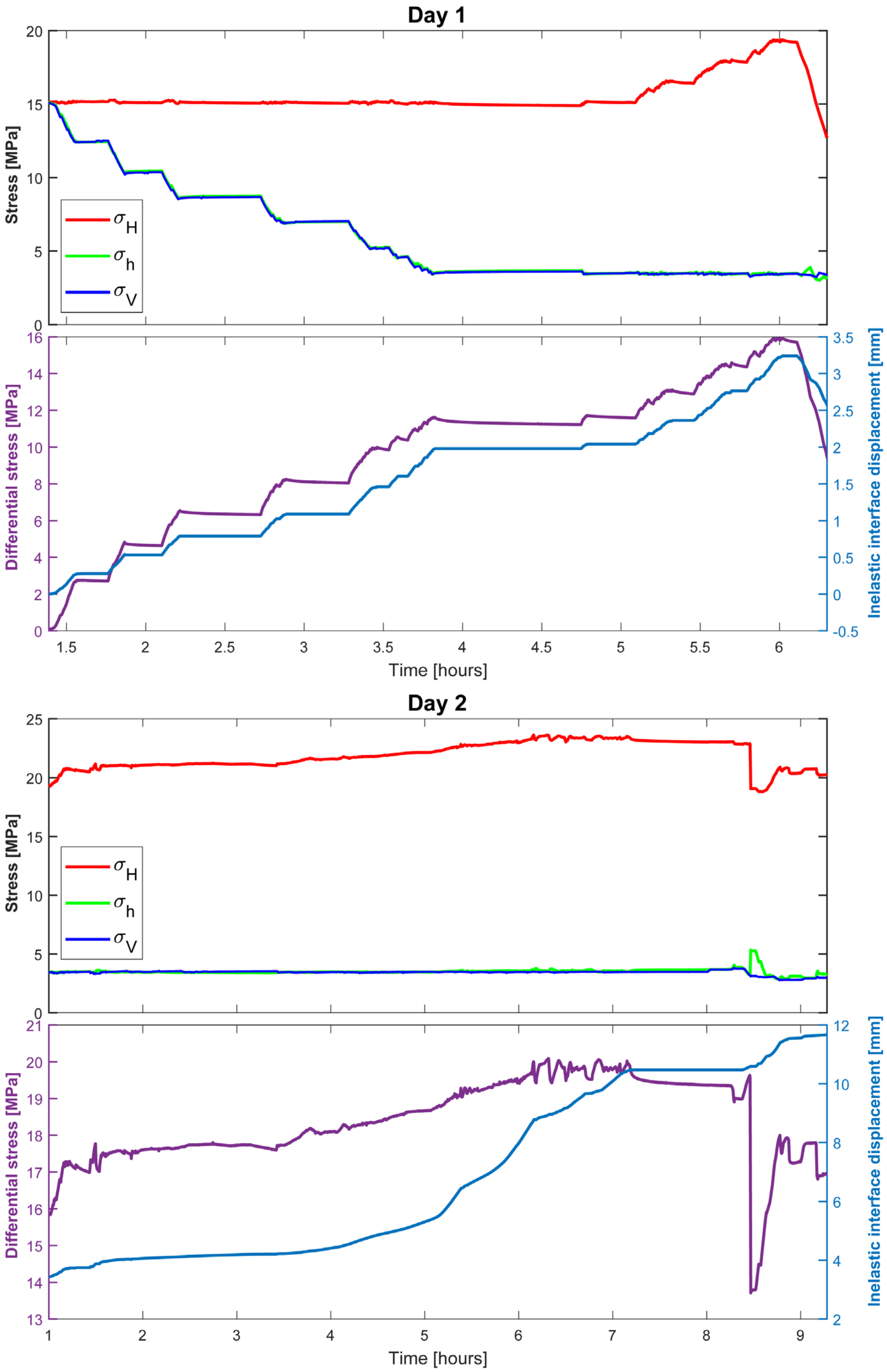

Fig. ES3: *Principal stresses, differential stress and displacement as function of time for days 1 and 2.*
