# Supplementary material for: Cubic-meter scale laboratory fault re-activation experiments to improve the understanding of induced seismicity risks
Source: Sci Rep. 2022 May 15;12:8015. doi: 10.1038/s41598-022-11715-6 (PMC9108097; doi:10.1038/s41598-022-11715-6)
Supplement: Supplementary file 1 — Supplementary Information 1. [file 41598_2022_11715_MOESM1_ESM.pdf]

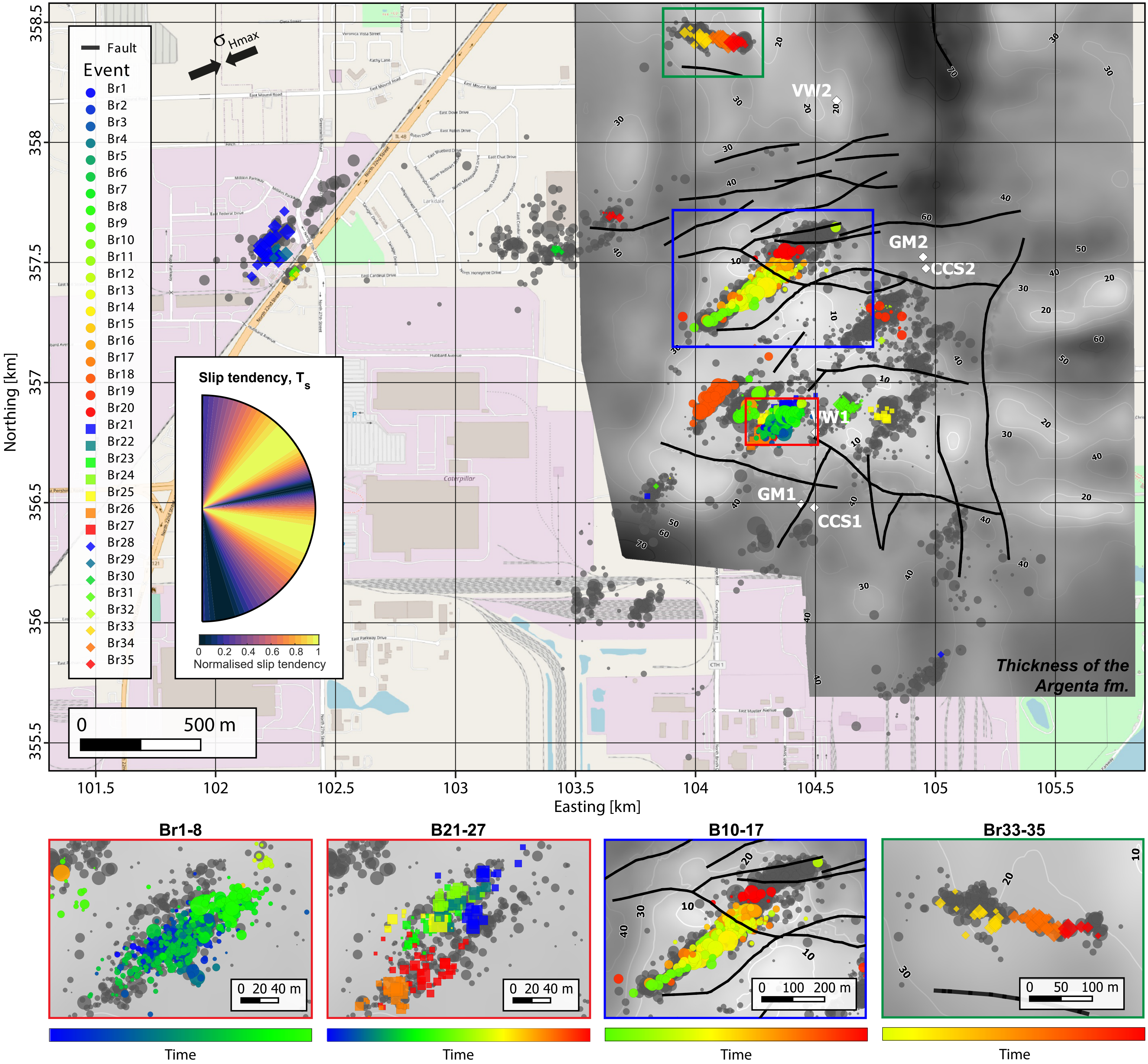

Fault
Event
Br1
Br2
Br3
Br4
Br5
Br6
Br7
Br8
Br9
Br10
Br11
Br12
Br13
Br14
Br15
Br16
Br17
Br18
Br19
Br20
Br21
Br22
Br23
Br24
Br25
Br26
Br27
Br28
Br29
Br30
Br31
Br32
Br33
Br34
Br35
σ Hmax
Slip tendency, T_s
0 0.2 0.4 0.6 0.8 1
Normalised slip tendency
0 500 m
VW2
GM2
CCS2
W1
GM1
CCS1
Thickness of the Argenta fm.
Northing [km]
Easting [km]
101.5 102 102.5 103 103.5 104 104.5 105 105.5
355.5 356 356.5 357 357.5 358 358.5
Br1-8
B21-27
B10-17
Br33-35
0 20 40 m
0 100 200 m
0 50 100 m
Time
